# Editorial: Synaptic Diseases: From Biology to Potential Therapy

**DOI:** 10.3389/fnsyn.2022.846099

**Published:** 2022-03-09

**Authors:** Hansen Wang, Rita Balice-Gordon

**Affiliations:** ^1^Leslie Dan Faculty of Pharmacy, University of Toronto, Toronto, ON, Canada; ^2^Muna Therapeutics, Copenhagen, Denmark

**Keywords:** synaptic diseases, synaptic disorders, synapse, brain, synaptic plasticity, neurodevelopmental diseases, neurodegenerative diseases, translational medicine

## Introduction

Mutations in genes encoding synaptic or synapse-related proteins that affect the structure and/or function of synapses are responsible for various forms of synaptopathies, including neurodevelopmental, neurodegenerative and psychiatric diseases (Lepeta et al., 2016; Lima Caldeira et al., 2019; Bonnycastle et al., 2021; Germann et al., 2021). Understanding how disease causing genes affect synapse structure and function, and cause circuit and behavioral dysfunction, has been a focus of neuroscience research over several decades. Many challenges remain to be addressed, from identifying rare disease-associated genes, defining the molecular and cellular mechanisms by which the genetic mutations confer disease risk and manifest as phenotypes, understanding how these mutations affect circuit function, plasticity and behavior, to whether therapeutic interventions can restore function. Studying the pathophysiologic mechanisms underlying synaptopathies will lead to a better understanding of the molecular and cellular mechanisms that govern normal nervous system function, and may eventually help to discover impactful therapeutics (Wang and Doering, 2015; Lepeta et al., 2016; Lima Caldeira et al., 2019; Carroll et al., 2021).

This Research Topic has focused on advances in studying common synaptopathies, collecting 31 research and review articles ranging from new insights into fundamental synapse biology to potential therapeutic strategies. Here we summarize each of these articles as a guide to the Research Topic, and highlight the many new research questions stimulated by the work.

## Papers In This Research Topic

### Synapse and Circuit Biology: Neuropathologies

Understanding how neural circuits and synaptic connectivity are established and modulated in the brain is essential for elucidating the mechanisms of neurological disorders. Dendritic spines that compartmentalize synapses to fine-tune synaptic strength are critical for neuronal connectivity (Koleske, 2013; Mironova and Giger, 2013; Maiti et al., 2015; Lepeta et al., 2016; Germann et al., 2021). Xiong et al. reported a method for delivering cell-impermeable compounds into neurons based on Spatially resolved NAnoparticle-enhanced Photoporation (SNAP), to efficiently label selected individual neurons and their dendritic spines in cultured neuronal cells, holding promise for studying neuronal connectivity at disease conditions.

Synaptic assembly is regulated by secreted cell-cell signaling molecules, such like Wnts, which are involved in the development and maintenance of nervous system. Shi et al. discovered that Wnt-2 is required for synaptic development in *C. elegans* and provided genetic evidence for the roles of Wnt-2 in promoting synaptogenesis, providing further insights into Wnt-related neurological disorders. The interdependent proceses of cell proliferation and differentiation were studied by Micheli et al. who investigated the possibility to restore the impaired terminal differentiation of progenitor cells in hippocampal dentate gyrus through induction of their proliferation or differentiation. They demonstrated that NeuroD2 or the silencing of Id3 could activate the differentiation of neurons in dentate gyrus, complementing the impairment of differentiation due to lack of Tis21. The study highlighted how the differentiation of dentate gyrus neurons is genetically modulated and that neurogenic stimuli to amplify neural stem/progenitor cells may not be enough to regulate their differentiation. Neurotransmitters are important for the formation and maturation of synaptic circuits during brain development. Ojeda and Avila summarized studies on the roles of neurotransmitters in early cortex development and maturation of reprogrammed neurons, highlighting the ways that research in this field has helped to understand the mechanisms for brain development and to develop potential treatments for neurodevelopmental disorders.

Arf1 GTPase activating protein (AGAP1) can interact with the vesicle-associated proteins and is a susceptibility candidate for autism spectrum disorder (ASD) and schizophrenia. Arnold et al. reported that AGAP1 is localized to axons, dendrites, dendritic spines and synapses, colocalizing with the markers of early and recycling endosomes; up- or downregulation of AGAP1 could affect both neuronal endosomal trafficking and the morphology of dendritic spines, supporting the roles of AGAP1 in these processes. The *DTNBP1* gene is known to be associated with neurodevelopmental disorders. They further revealed that AGAP1 is selectively reduced in the knockout mice of *DTNBP1*. These findings suggested that AGAP1 act downstream of DTNBP1 as a regulator of endosomal trafficking to get involved in the pathogenesis of neurodevelopmental disorders through modulating dendritic spines and synapses.

Semaphorins participate in neuronal development and axon guidance. Zwarts et al. studied the roles of Sema-1a in mushroom body (MB) development in the Drosophila brain. By loss- and gain-of-function approaches, they found that the MB axons show lobe and sister branch-specific Sema-1a signal, that controls the axon outgrowth and guidance; these effects are regulated by integrating MB intrinsic and extrinsic Sema-1a signal pathways; neuron-glia interaction is involved in Sema-1a dependent beta-lobe outgrowth.

Parallel fiber-Purkinje cell synapses represent an example of maximal signal divergence in cerebellar cortex. Hoxha et al. reviewed the state of current knowledge about the regulation, plasticity and pathophysiology of parallel fiber-Purkinje cell synapses, and emphasized that several different structural and other methodological approaches need to be applied to develop a fuller understanding of the pathophysiology of diseases involving this key circuit in the cerebellum.

The function of synapses depends on the microtubule (MT) cytoskeleton. The dynamics of MT are controlled by MT-associated proteins, including the MT-stabilizing protein Tau. Mutations of the Tau-encoding *MAPT* gene cause a subset of neurodegenerative diseases, termed tauopathies. Verstraelen et al. studied the effect of targeted perturbation of MT stability and found that treatment with MT-stabilizing or -destabilizing drugs disrupted morpho-functional connectivity in networks of primary hippocampal neurons in a reversible manner. While overexpression of *MAPT* caused defects in connectivity, accompanied by altered dynamics of MT and increased resistance to pharmacological depolymerization of MT, overexpression of a *MAPT* with P301L mutation in the MT-binding domain led to fewer deficits, thus connecting neuronal connectivity with MT binding affinity of Tau. These findings indicate that pharmacological tuning of MT stability could positively affect neuronal network connectivity and may have therapeutic potential for neurodegenerative disorders.

The cell surface adhesion molecule neurofascin plays dual role in the establishment of axonal domains from both glial and neuronal interface. Taylor et al. used spatially and temporally targeted deletion of neurofascin in neurons or in both neurons and myelinating glia in postnatal mice and revealed that the maintenance and health of nodes and axons are correlated to neuron-specific isoform of neurofascin NF186 in the nodal complex and the presence of auxiliary paranodes. Sirtuins (SIRTs) are involved in the genetic modulation of energy metabolism, stress response and neurodegeneration. SIRT1 and SIRT6 are nuclear and share similar activities and localizations at both cellular and brain tissue levels. Tang emphasized that understanding the differences and crosstalk between SIRT1 and SIRT6 will be essential for further elucidating the potential of SIRTs as targets for treating neurodegenerative diseases.

Autoantibodies against NMDARs in the cerebrospinal fluid (CSF) from patients of anti-NMDAR encephalitis could be pathogenic. Blome et al. further demonstrated that anti-NMDAR containing CSF impairs the long-term potentiation (LTP) at the associational-commissural fiber-CA3 synapse of hippocampus. The different inhibition of LTP at this synapse in comparison to the mossy fiber-CA3 synapse suggests the specific effect and underlines the pathophysiological roles of NMDAR-antibodies.

### Synapse and Circuit Biology: Neurodevelopmental Disorders

Alterations in neurite arborization and dendritic spine morphology are hallmarks of almost all neurological conditions. Many of autism risk genes converge into similar signaling pathways to regulate synapse formation and function. Lin et al. reviewed the current knowledge about the autism risk genes that affect the neuronal structure connectivity, pointing out that investigating the neuronal structure and function affected by mutation of those genes may help to develop therapies for ASD. Accumulating evidence has underscored the roles of inhibitory synapse formation, specialization, and function in ASD and other neurodevelopmental disorders. Ali Rodriguez et al. reviewed the mechanisms underlying the dysfunctions of inhibitory synapses in neurodevelopmental disorders, highlighting those common alterations inhibitory synapses observed in neurodevelopmental disorders.

Fragile X Syndrome (FXS), the most common form of intellectual disability, is a primary cause of autism. Many groups have worked on new therapeutic approaches for FXS over the last decade (Wang, 2015; Wang et al., 2015; Bagni and Zukin, 2019). Castagnola et al. reviewed the signaling pathways that underlie the physiopathology of FXS and summarized FXS treatment strategies to modulate these pathways with an emphasis on those shared with other synaptic disorders. The impairment of mitochondrial and oxidative challenge have been considered to facilitate the progression of Rett syndrome (RTT). Previous work has shown the benefits of acute treatment with the vitamin E-derivative Trolox in a RTT mouse model. Janc et al. reported results of a preclinical study to assess the therapeutic effects of systemic Trolox administration. They found *in vivo* Trolox treatment partially ameliorated several disease phenotypes, including lipid peroxidation, synaptic short-term plasticity, hypoxia tolerance and environmental exploration behaviors, supporting partial benefits of vitamin E-derivative based pharmacotherapy.

RTT is caused by mutations in the transcription factor methyl-CpG-binding protein 2 (MeCP2). MeCP2 deficient mice recapitulate irregular breathing phenotypes of RTT patients (Banerjee et al., 2019; Sandweiss et al., 2020). Vogelgesang, Niebert, Renner, et al. reported an altered expression of G-protein-coupled serotonin receptor 5-ht5b in the brainstem of these RTT mice. Vogelgesang, Niebert, Bischoff, et al. generated double knockout mice (*Mecp2*^−/*y*^;*Htr5b*^−/−^) and found that the breathing rate and the number of pauses in double knockout mice were indistinguishable from wild-type mice, supporting the roles of 5-ht5b receptors in the breathing phenotypes of MeCP2 deficient mice. The mutations of cyclin-dependent kinase-like 5 (CDKL5) are found in severe forms of neurodevelopmental disorders, e.g., the Hanefeld variant of RTT (CDKL5 disorder). Pizzo et al. investigated the cellular mechanisms for visual defects in CDKL5 disorder by examining the organization of the primary visual cortex (V1) in *Cdkl5*^−/*y*^ mice. They showed that the shift of excitation/inhibition balance caused by the disruption of V1 cellular and synaptic organization likely underlies the visual deficits in the disorder.

Loss of the maternally expressed *UBE3A* gene cause Angelman syndrome. The *Ube3a* mouse model recapitulates many of the symptoms seen in Angelman syndrome, showing the presence of synaptic deficits. Wang et al. investigated the roles of UBE3A in the auditory system and found that principal neurons in the medial nucleus of the trapezoid body (MNTB) of *Ube3a* mice show the hyperpolarization in resting membrane potential, the increase of action potential (AP) amplitude and decrease of AP half width. Furthermore, both the pre- and post-synaptic AP in the calyx of Held synapses of *Ube3a* mice display a faster recovery from spike depression. Additionally, the increase in axon initial segment length was found in the MNTB principal neurons of *Ube3a* mice, providing a potential base for those gain-of-function changes. These findings support that UBE3A is critical for controlling synaptic transmission and excitability at excitatory synapses. The post-synaptic adhesion molecule Netrin-G ligand-1 (NGL-1), encoded by Lrrc4c, is implicated in various brain diseases. Choi et al. demonstrated that *Lrrc4c*^−/−^ mice display the hyperactivity and anxiolytic-like behaviors, and impairment of spatial and working memory, but normal memory in object-recognition and social interactions. Neurons in several brain areas show distinct dysfunctions in excitatory synaptic transmission and intrinsic neuronal excitability. These data indicate that NGL-1 is important for synapse properties and excitability of neurons, as well as higher brain function.

### Synapse and Circuit Biology: Modulators of Behavioral Dysfunction in Psychiatric Diseases

Cholinergic hypofunction is related to decreased attention and cognitive deficits in the central nervous system, and enhancement of cholinergic neurotransmission has been proposed as a therapeutic approach to ameliorate cholinergic hypofunction in several neurological conditions. High affinity choline uptake into acetylcholine -synthesizing neurons is mediated by the choline transporter (CHT). Choudhary et al. utilized novel screening techniques to reveal both positive and negative modulation of CHT using literature tools. They identified a number of novel active and structurally distinct molecules that could be used as tools for further exploring CHT biology or as a starting point for future medicinal chemistry.

Ketamine can cause psychotic episodes and is used in animals for pharmacological model of psychotic-like behavior. Lisek et al. showed that ketamine-mediated inhibition of plasma membrane calcium pump, through decreasing total calcium clearing potency, might locally raise cytosolic calcium to promote excessive glutamate release. This work suggests a novel mechanism for psychogenic action of ketamine.

Stress could induce neuronal structure changes in the limbic system. These structural changes further lead to the development of stress-induced psychopathologies including depressive disorders. Csabai et al. examined synaptic contacts in rats subjected to depression and found that stress could reduce the number of synapses and myelinated axons in the deeper cortical layers, while synapse membrane lengths increased. The data suggest that neurons in infralimbic cortex have reduced cortical neuronal network connectivity, potentially leading to impaired functioning of impaired functioning of this brain area. Chronic restraint stress induces depressive-like behaviors, anxiety and reduced dendritic spine density in hippocampal neurons. Pacheco et al. found that chronic stress promoted expression of immediate early genes, triggered a reduction in PSD-95 in both dorsal and ventral hippocampal areas, and differently affected AMPA and NMDA receptor (NMDAR) subunits in these hippocampal areas.

Binge drinking is known as the most common form of alcohol abuse. Little is understood about the biobehavioral effect of binge-drinking during the neurodevelopmental period of adolescence. Using a mouse model of binge drinking, Lee et al. demonstrated that adolescents are resilient to many negative effects of early alcohol withdrawal, and the reduced sensitivity to the negative effects of binge drinking might facilitate more alcohol intake among adolescent drinkers.

### Synapse-Centric Therapeutic Strategies for Neurodegenerative Diseases

The pathologies of Synapses and mitochondria are the early events in the progression of Alzheimer's disease (AD). Zhang, Zhao, et al. found that geniposide alleviated β-amyloid (Aβ)-induced abnormalities of axonal mitochondria by increasing the density and length of axonal mitochondria and promoting the motility and trafficking of mitochondria in cultured hippocampal neurons, consequently attenuating synaptic damage in neurons and AD mice. This work provided new insights into the effect of geniposide on neuronal and synaptic function in the presence of Aβ. Aβ is produced by beta-secretase 1 (BACE1)-mediated enzymatic cleavage of the amyloid precursor protein, supporting BACE1 as a therapeutic target in AD. Villamil-Ortiz et al. found that RNA interference against BACE1 treatment induced the recovery of cognitive function and restored fatty acid composition and lipid metabolism in hippocampus of triple transgenic AD mice, suggesting that the restoration of phospholipids composition by BACE1 silencing could help the recovery of cellular homeostasis and cognitive function of AD mice. One of the earliest events in AD is synaptic loss caused by soluble oligomeric forms of the Aβ peptide. Pannexin 1 (Panx1) channels have been known to modulate excitatory synaptic plasticity under physiological conditions. Flores-Munoz et al. observed an age-dependent increase of Panx1 expression that correlates with increased levels of Aβ in hippocampus of transgenic AD mouse model. An exacerbated Panx1 activity was also observed at the basal condition and in responding to the activation of glutamate receptors. Pharmacological inhibition of Panx1 activity did not affect neurodegenerative markers, but attenuated excitatory synaptic defects in hippocampal neurons of AD mice, indicating enhanced expression and activity of Panx1 contributes to Aβ-induced synaptic deficits in AD.

Homocysteine (HCY), an endogenous redox active amino acid that activates NMDARs, contributes to various forms of neurodegenerative diseases. Sibarov et al. found that HCY-induced NMDA receptor currents in neurons are mostly mediated by the synaptic type of GluN1/2A NMDARs. They also found that the toxicity of HCY may be controlled by desensitization of HCY-induced activation of GluN2B-containing extra-synaptic receptors. Together these data support the physiological role of HCY as an endogenous modulator of excitatory neurotransmission.

Damage and mutations of Mitochondrial DNA (mtDNA) are involved in the progressive loss of retinal ganglion cells (RGCs) in glaucoma animal model. Zhang, Gao, et al. showed that high pressures can directly cause alterations of mtDNA, resulting in the dysfunction of mitochondria and the death of RGCs.

### New Insights Into Roles of Microglia in Neurodegenerative Diseases

Microglia prune synapses of neurons and regulate the plasticity and function of synapses. Disruption of microglia-synapse interaction can cause the loss and dysfunction of synapses, and consequently cognitive defect in AD (Bar and Barak, 2019; Bartels et al., 2020; Subramanian et al., 2020). Understanding of the diversity of microglia has grown in recent years, reflecting the use of single-cell multi-omic profiling in animal models and human post-mortem brain samples. Wang summarized the current studies of the heterogeneity of microglia and modulation of microglial phenotypes in the brain of both AD mouse models and patients using single-cell technologies. Defining the functions of distinct microglia states will provide further insights into the pathological roles of microglia and may help to discover relevant therapeutic targets for AD.

Disease associated microglia (DAM) were first reported by a study using the 5xFAD mouse model of AD (Keren-Shaul et al., 2017). DAM are AD-associated phagocytic cells and are conserved in mice and human (Grubman et al., 2019; Mathys et al., 2019; Zhou, 2020; Gerrits et al., 2021). Because DAM have the potential to restrict neurodegeneration, modulating this phenotype may have therapeutic relevance for AD and other neurodegenerative diseases. Wang reviewed recent studies on pharmacological modulation or reprogramming of DAM, such as blocking microglia-specific checkpoints, that suggest new therapeutic intervention strategies for AD.

## Concluding Remarks

In summary, this Research Topic has highlighted the diversity of research on pathways, mechanisms and potential therapeutic strategies for neurological diseases over the last 6 years. The Research Topic of research articles and reviews highlights efforts from research teams worldwide in this dynamic field. We hope that this collection will be informative to researchers and will encourage follow-on studies to support the advancement of new knowledge, and ultimately therapeutic interventions for synaptopathies which represent significant unmet medical need around the globe.

## Author Contributions

HW drafted the manuscript. HW and RB-G edited the manuscript, contributed to the Research Topic, and approved the publication of this Editorial.

## Conflict of Interest

RB-G was employed by Muna Therapeutics. The remaining author declares that the research was conducted in the absence of any commercial or financial relationships that could be construed as a potential conflict of interest.

## Publisher's Note

All claims expressed in this article are solely those of the authors and do not necessarily represent those of their affiliated organizations, or those of the publisher, the editors and the reviewers. Any product that may be evaluated in this article, or claim that may be made by its manufacturer, is not guaranteed or endorsed by the publisher.
